# Coiling of the Internal Carotid Artery is Associated with Hypertension in Patients Suspected of Stroke

**DOI:** 10.1007/s00062-020-00892-4

**Published:** 2020-03-18

**Authors:** Josephus L. M. van Rooij, Richard A. P. Takx, Birgitta K. Velthuis, Jan Willem Dankbaar, Pim A. de Jong, the DUST investigators

**Affiliations:** Department of Radiology, University Medical Center Utrecht, Heidelberglaan 100, 3584 CX Utrecht, The Netherlands

**Keywords:** Tortuosity, Coiling, Internal carotid artery, Dolichoarteriopathy, Hypertension

## Abstract

**Purpose:**

The etiology of coiling (i.e. severe elongation) of the extracranial part of the internal carotid artery (ICA) is poorly understood with the proposed etiology being congenital, atherosclerotic or hypertension. The objective was to investigate the association of coiling with hypertension, carotid artery atherosclerosis and other cardiovascular risk factors.

**Methods:**

A case control study was performed in patients suspected of stroke, with (cases) or without (controls) coiling of the ICA determined on compute tomography angiography (CTA). Baseline characteristics included age, gender, hypertension, diabetes, smoking and hypercholesterolemia. Coiling of the ICA and atherosclerotic plaque at the carotid bifurcation were assessed on CTA. Logistic regression analyses were conducted.

**Results:**

Coiling was identified in 108 patients with a median age of 71 years. Cases were compared with 256 controls with a median age of 69 years. Hypertension was present in 63% of the patients with coiling compared to 51% in the control group. Univariable analysis showed that hypertension was significantly associated with coiling, with an odds ratio of 1.65 (95% confidence interval (CI) 1.04–2.61, *p* = 0.034). Multivariable analysis corrected for age and sex resulted in an odds ratio of 1.71 (95% CI 1.05–2.80, *p* = 0.032), while correcting for atherosclerotic plaque at the bifurcation yielded an odds ratio of 1.63 (95% CI 1.00–2.66, *p* = 0.049). Age and atherosclerotic plaque were not significantly associated with coiling.

**Conclusion:**

The main finding of this study was the significant association of hypertension with coiling of the ICA and the absence of an association with age, plaques and atherosclerotic risk factors other than hypertension.

## Introduction

Coiling (i.e. severe elongation) of the internal carotid artery (ICA) is a rare morphologic alteration in the course of the artery. The extracranial part of the ICA runs from the carotid bifurcation up to the entry into the petrous segment of the temporal bone [1]. The anatomical course of the extracranial part of the ICA can differ, varying from a straight course in most cases to elongation in some [1]. This elongation has been described as dolichoarteriopathy and is classified in three categories: ranging from severe (coiling), moderate (kinking) to mild (tortuosity) [1]. Coiling has been found in 3–4% of a healthy population and has been associated with cerebrovascular insufficiency [2]. To identify associated factors the greatest contrast for power reasons between controls and the most severe dolichoarteriopathy (i.e. coiling) were introduced. Different theories have been proposed regarding the etiology of coiling. Coiling has been described as a persisting embryological state [3, 4], which is usually bilaterally present [5]. Advancing age may also contribute to the development of coiling through degradation and fragmentation of intramural elastin of the arterial wall [6]. Another hypothesis is that hypertension is a possible cause for the development of coiling [6]. Hypertension can lead to arterial vessel lengthening due to an imbalance between forces to the vessel wall [7]. This can result in an increase in the diameter and loss of stiffness of large arterial vessels, resulting in stretching of circumferential smooth muscle cells of the tunica media [8]. Finally, atherosclerotic remodeling could result in coiling of the arteries through hemodynamic abnormalities [9, 10]. The objective of this article is to investigate the association of coiling with hypertension, carotid atherosclerosis and other cardiovascular risk factors.

## Material and Methods

### Study Design

This was a retrospective analysis from data acquired for the Dutch Acute Stroke study (DUST) from the period 2009–2014 [11]. This multicenter prospective, observational study included 1393 patients who underwent computed tomography angiography (CTA) in the diagnostic work-up of a possible acute ischemic stroke. Inclusion criteria were: I) age of 18 years and older; II) the time of onset of acute neurological deficits was less than 9h and III) a score of two or higher on the National Institutes of Health Stroke Scale (NIHSS) or 1 if an indication for intravenous tissue plasminogen activator (itPA) treatment was present. Exclusion criteria were a diagnosis other than ischemia on non-contrast CT scanning, renal failure or contrast allergy. Informed consent of patients or their family was obtained unless the patient had died, in which case the need for informed consent was waived. More details regarding methods and study protocol have been previously described [11]. Patients of the DUST study were selected to form a case control study, comparing a population with coiling (cases) to a random sample of patients with no coiling (controls), with a ratio of approximately 1:2. Patients with kinking or tortuosity were excluded.

### CT Imaging Protocol

Images were obtained using 40–320 detector row CT scanners (Philips, Best, the Netherlands; Siemens Healthineers, Erlangen, Germany; GE Healthcare, Milwaukee, WI, USA; Toshiba, Medical Systems, Tochigi, Japan). The scan was obtained from the aortic arch to the cranium vertex. Non-ionic intravenous contrast was injected (50–70 ml) with a flow of 6 ml/s followed by saline (40 ml) with also a flow of 6 ml/s. For each patient the scan delay was calculated from time to peak arterial enhancement on CT perfusion or with contrast bolus tracking in the aortic arch [11].

### Assessment of Risk Factors and Atherosclerotic Plaque

Patients characteristics were obtained at admission and included age, hypertension, diabetes mellitus, hypercholesterolemia and smoking habits. Other cardiovascular risk factors, such as body mass index and a family history of cardiovascular disease were not systematically included. The presence of atherosclerotic plaque (calcified, mixed or non-calcified) was evaluated in the left and right ICA by an experienced radiologist.

### Selection of Cases and Controls

**Fig. 1:**
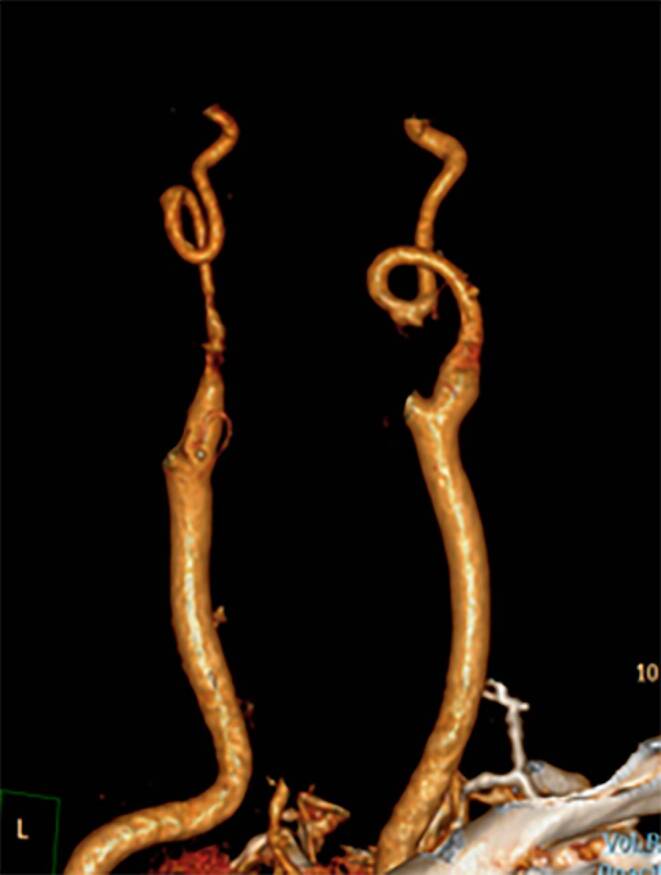
3D reconstructed CT image demonstrating coiling of both internal carotid arteries in a circular configuration

First a trained medical student under supervision of a radiologist evaluated all scans and preselected 146 cases that were suspected of coiling. In a second step these were formally evaluated according to the criteria by Metz et al. [12] and Weibel et al. [13] who described coiling as an extended elongation of the artery in a restricted space, resulting in a circular or exaggerated S‑configuration of the artery (Fig. 1). This second round was done by a second trained medical student (JvR) under supervision of two radiologists (JWD, PDJ) and difficult cases were discussed in consensus.

### Statistical Analysis

For clinical risk factors as well as CT measures normality of the data was determined using quantile-quantile plots. Normally distributed data were reported as mean (95% confidence interval, CI) and non-normally distributed data as median (25th percentile [P25] and 75th percentile [P75]). Binary variables were reported as frequency (percentage). Logistic regression models were used with and without the addition of potential confounders as covariates (sex and age, atherosclerotic plaque) to calculate OR and 95% CI. A *p*-value <0.05 was deemed statistically significant. The SPSS random number generator was used for selection of the controls [14]. Statistical analyses were performed using SPSS version 24.0 (IBM Corp, Armonk, NY, USA).

## Results

### Patient Selection

**Fig. 2:**
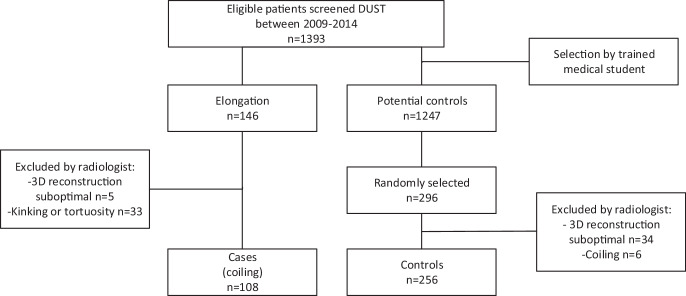
Study population selection flow chart

Of the 146 patients who were identified as having marked elongation of the ICA, 5 scans were excluded because evaluation and 3D reconstruction was suboptimal due to moderate CTA image quality. Of the remaining 141 patients, 33 patients did not fulfil the coiling criteria but had kinking or tortuosity and were excluded for final analysis. This resulted in a total of 108 patients having coiling, of which 73 patients had bilateral coiling and 35 patients had unilateral coiling. Of the 296 randomly selected cases where no anomaly was observed in the preselection, 34 patients were excluded due to suboptimal evaluation/3D reconstruction. Of the remaining 262 patients, 6 scans were excluded from the current study for having coiling, resulting in 256 controls (Fig. 2).

### Baseline Characteristics

Of the patients with coiling, 39% were female versus 46% of the patients without coiling. Median age of the cases (71 years, range 62–77 years) was comparable to the age of the control group (median age of 69 years, range 57–78 years). A history of hypertension was described in 63% of the study group and in 51% of the control group (*p* = 0.033). The groups were comparable regarding the other cardiovascular risk factors and the prevalence of plaques at the bifurcation (Table 1).

**Table 1:** Baseline characteristics of the study population in those with coiling and controls

	Coiling (*n* = 108)	Controls (*n* = 256)	*P*-value
*Age in years median (P25–P75)*	71 (37–96)	69 (57–78)	0.743
*NIHSS median (P25–P75)*	6 (3–11)	6 (3–11)	0.694
*Female sex, n (%)*	42 (39%)	118 (46%)	0.206
*Diabetes, n (%)*	15 (14%)	45 (18%)	0.389
*Hypertension, n (%)*	68 (63%)	129 (51%)	0.033
*Hypercholesterolemia, n (%)*	39 (37%)	88 (36%)	0.767
*Smoking, n (%)*^*a*^	67 (66%)	150 (65%)	0.844
*Presence of atherosclerotic plaque in the ICA, n (%)*	81 (76%)	192 (75%)	0.821

^a^Current/previous smoking

*NIHSS* National Institutes of Health Stroke Scale, *ICA* internal carotid artery

### Odds Ratios for Having Coiling

When comparing the group of coiling versus controls, a history of hypertension was found to be significantly associated with the presence of coiling in univariable analysis, which resulted in an OR of 1.65 (95% CI 1.04–2.61, *p* = 0.034). Age, gender, other cardiovascular risk factors and the measured plaques were not significantly associated with the presence of coiling. Hypertension remained significantly associated with coiling after adjusting for age and sex (OR 1.71; 95% CI 1.05–2.80, *p* = 0.032) and after adjusting for the presence of atherosclerotic plaque at the bifurcation (OR 1.63; 95% CI1.00–2.66, *p* = 0.049). Detailed univariable and multivariable OR are listed in Table 2.

**Table 2:** Uni- and multivariable odds ratios on the association of coiling of the ICA and possible risk factors

	Univariable	*P*-value	Age and sex corrected	*P*-value	Atherosclerotic plaque corrected	*P*-value	Age, sex and atherosclerotic plaque corrected	*P*-value
Age in years	1.01 (0.99–1.02)	0.481	NA	NA	1.01 (0.99–1.03)	0.423	NA	NA
NIHSS	1.00 (0.96–1.04)	0.941	1.00 (0.96–1.04)	0.904	1.00 (0.96–1.04)	0.931	1.00 (0.96–1.04)	0.954
*Female sex*	0.74 (0.47–1.18)	0.744	NA	NA	0.73 (0.46–1.17)	0.734	NA	NA
*Diabetes*	0.76 (0.40–1.43)	0.390	0.72 (0.38–1.37)	0.313	0.72 (0.37–1.38)	0.321	0.70 (0.36–1.35)	0.286
*Hypertension*	1.65 (1.04–2.61)	0.034	1.71 (1.05–2.80)	0.032	1.63 (1.00–2.66)	0.049	1.68 (1.01–2.78)	0.044
*Hypercholesterolemia*	1.07 (0.67–1.73)	0.767	1.03 (0.63–1.67)	0.911	0.99 (0.61–1.62)	0.974	0.96 (0.59–1.57)	0.865
*Smoking*^*a*^	1.05 (0.64–1.72)	0.844	1.01 (0.61–1.69)	0.956	1.01 (0.62–1.67)	0.959	0.99 (0.59–1.67)	0.976
*Presence of atherosclerotic plaque in the ICA*	1.06 (0.63–1.81)	0.821	0.90 (0.49–1.67)	0.744	NA	NA	NA	NA

^a^Current/previous smoking

*NIHSS* National Institutes of Health Stroke Scale, *ICA* internal carotid artery, *NA* not applicable

## Discussion

The main finding of this study was the significant association of hypertension with coiling of the ICA and the absence of association with age, plaques and atherosclerotic risk factors other than hypertension. These results support the hypothesis that hypertension is linked to coiling although causality cannot be concluded with certainty based on the cross-sectional data. It maybe that coiling is caused by hypertension, but it may also be that coiling is a preventive mechanism of the human body to the effects of hypertension. Hypertension is detrimental for the brain and a cause of stroke. Although, coiling maybe an advantageous mechanism to reduce the effect of hypertension on the brain.

Previous investigators showed an association between hypertension and a combined group of patients with coiling, tortuosity and kinking in smaller studies or imaging techniques that are less reliable than CT [15–17]. Although this study only included coiling, kinking and tortuosity may be due to a similar mechanism as coiling. In 1998 Del Corso et al. [9] investigated a study group of 469 patients (median age 66 years, 53% men) using Doppler sonography in the diagnostic work-up for neurologic symptomatology or for general vascular assessment. The main outcome was that carotid abnormalities (i.e. a combined group of coiling, kinking and tortuosity) were significantly associated with hypertension in univariable analysis (52% versus 39%, *p* < 0.001). Nevertheless, no subanalysis was performed for coiling alone and no multivariable analysis was conducted to adjust for potential confounders. Pancera et al. [16] also found an association between kinking and hypertension in 590 patients (38% versus 28%, *p* = 0.02) using ultrasound without multivariable adjustment. In contrast, Beigelman et al. [3], also using ultrasound and no multivariable adjustment, reported no significant association between hypertension in patients with kinking and coiling as a combined group versus patients with no elongation of the carotid arteries in 885 patients.

In the present cohort an association between either age or the presence of atherosclerosis lesions was not observed. One possible explanation for the lack of an association with age can be that the cohort consisted of older patients. The cohort was also biased towards atherosclerotic patients as they were referred for possible stroke.

A strength of the current study is that multivariable analysis was performed while in most reports only univariable analysis was performed. Coiling was also strictly defined while others investigated a combined group of kinking, tortuosity and coiling. In addition, CTA is superior to Doppler ultrasonography used in other studies to evaluate the distal part of the extracranial carotid artery (>4 cm beyond the carotid bifurcation) [18]. One main limitation of the current study is that a selected population of patients suspected for having a stroke was used. This results in a relatively high mean age of the cohort with a high incidence of hypertension and other cardiovascular risk factors. A second limitation is that in any observer study variation between readers exists and in the random controls 2% had coiling according to the observers and were exclude from the analysis. A third limitation is that controls were randomly selected and the associations may have been stronger if controls with kinking and tortuosity were also excluded. Finally, since a cross-sectional case control observational design was applied causality cannot be inferred.

## Conclusion

This study demonstrated that hypertension is significantly associated with coiling of the extracranial part of the ICA. No associations were found with age, atherosclerosis and other cardiovascular risk factors. This supports the theory that hypertension is associated to this variation in carotid shape.

## Abbreviations

DUST: Dutch Acute Stroke study
ICA: Internal carotid artery
OR: Odds ratio
rtPA: recombinant tissue-type plasminogen activator
